# Cross Mol: cross-modal mask-predict pre-training for 3D molecular data

**DOI:** 10.1093/bioinformatics/btag702

**Published:** 2026-09-23

**Authors:** Kangjie Zheng, Junwei Yang, Siyu Long, Wei Ju, Wei-Ying Ma, Hao Zhou, Ming Zhang

**Affiliations:** State Key Laboratory for Multimedia Information Processing, Peking University, Beijing 100871, China; School of Computer Science, Peking University, Beijing 100871, China; PKU-Anker LLM Lab, Peking University, Beijing 100871, China; State Key Laboratory for Multimedia Information Processing, Peking University, Beijing 100871, China; School of Computer Science, Peking University, Beijing 100871, China; PKU-Anker LLM Lab, Peking University, Beijing 100871, China; School of Artificial Intelligence, Nanjing University, Nanjing, Jiangsu 210023, China; School of Artificial Intelligence, Sichuan University, Chengdu, Sichuan 610065, China; Institute for AI Industry Research (AIR), Tsinghua University, Beijing 100084, China; Institute for AI Industry Research (AIR), Tsinghua University, Beijing 100084, China; State Key Laboratory for Multimedia Information Processing, Peking University, Beijing 100871, China; School of Computer Science, Peking University, Beijing 100871, China; PKU-Anker LLM Lab, Peking University, Beijing 100871, China

## Abstract

**Motivation:**

Self-supervised pre-training models for molecular data have demonstrated notable results across many downstream tasks. The inherent multimodal properties of molecules have also motivated efforts to capture information from different modalities. However, current multimodal molecular pre-training models usually treat these modalities as equal and independent, despite differences in their information content. Three-dimensional (3D) molecular structures generally contain finer-grained information than the simplified molecular-input line-entry system (SMILES), which primarily captures higher-level semantic information, such as molecular topology.

**Results:**

We designed a cross-modal mask-predict pre-training model, CrossMol, to capture semantic associations between modalities with unequal information volumes. The model completes missing 3D structure using higher-level semantic information from another modality, such as SMILES. This allows it to learn cross-modal associations and better understand fine-grained 3D structural information. We also introduce a reweighted distance prediction loss to improve the modelling of short-range structural information. Experiments show that CrossMol achieves large performance gains on multiple downstream molecular tasks, attaining state-of-the-art results.

**Availability and Implementation:**

The source code and training data are available at https://github.com/zhengkangjie/crossmol. The code is archived at https://doi.org/10.5281/zenodo.19653204.

## 1 Introduction

Pre-training for molecular representation learning has shown excellent results in various molecular tasks like drug discovery (Adelusi *et al.* 2022), predicting molecular properties (Liu *et al.* 2022b, Luo *et al.* 2022, Ju *et al.* 2023, Xia *et al.* 2023, Zhou *et al.* 2023, Yu *et al.* 2024), and reaction prediction (Gastegger *et al.* 2021). Initially, methods often used one-dimensional (1D) simplified molecular-input line-entry system (SMILES) formats (Honda *et al.* 2019, Wang *et al.* 2019, Zheng *et al.* 2025a) or two-dimensional (2D) graphs (Li *et al.* 2021, Lu *et al.* 2021) to represent molecules.

However, most current methods that use multimodal molecular data treat inputs from different modalities as if they are equally important, as shown in Fig. 1. This approach overlooks the differences in the amount and emphasis of information conveyed by each modality. 1D (SMILES) and 2D (molecular graphs) data mainly provide concise descriptions of atom types and chemical bonds, while three-dimensional (3D) molecular structures include fine-grained details such as atomic coordinates. These structural details allow models to capture additional molecular properties, making 3D data generally richer and more diverse than 1D and 2D data. Meanwhile, 1D and 2D data can be viewed as abstract, high-level representations of 3D structures, capturing molecular semantics in a simplified form. This suggests that instead of treating all modalities equally, models should focus on learning detailed 3D structures, guided by the abstract semantic information embedded in 1D and 2D modalities.

**Figure 1:**
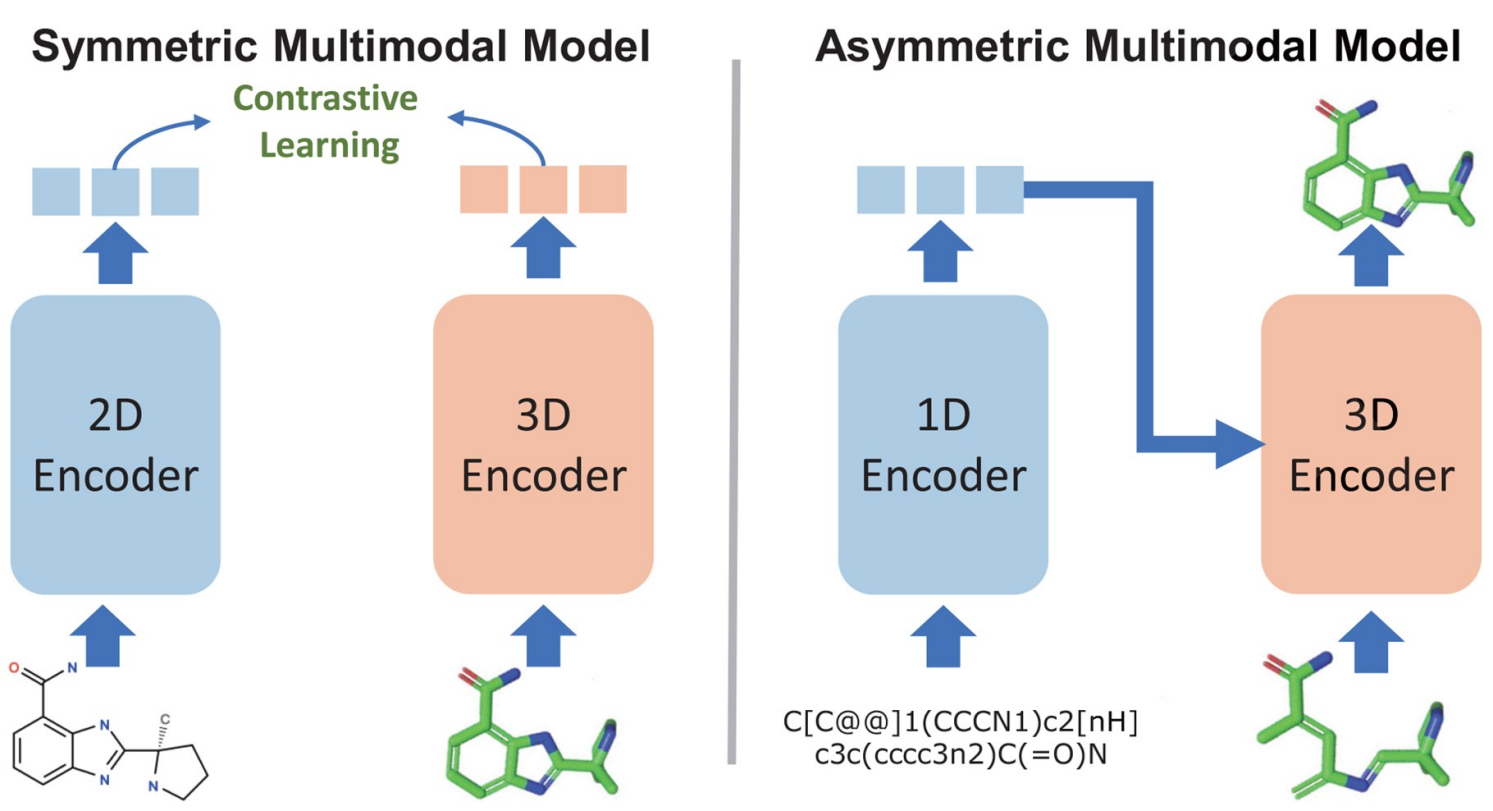
Differences between symmetric and asymmetric multimodal: in symmetric multimodal models, different modalities are treated equally, meaning they can be exchanged without any additional impact. In contrast, asymmetric multimodal models handle information from different modalities in a non-equivalent manner.

Given this, we propose that a fundamental principle in the design of multimodal molecular models should be to use the information from 1D (or 2D) modalities to enhance the understanding of 3D molecular structures, rather than treating the information from different modalities as equally independent inputs. A practical strategy is to input high-level data (e.g. 1D SMILES and 2D molecular graphs) and train the model to reconstruct full 3D structures using both this data and partial 3D inputs. This highlights the value of using high-level semantic cues to better exploit the fine-grained 3D information and improve model performance. In such an asymmetric design, 3D molecular data remains central, while 1D/2D modalities provide abstract representations to assist structural understanding.

To implement this, we propose the CrossMol model, which employs an Encoder-Decoder framework with a Cross-Modal Mask-Predict training objective to enhance 3D modelling. In summary, the contributions of this paper are threefold:

- We propose the first Encoder-Decoder-based multimodal fusion mechanism that fully leverages 1D and 2D data to enhance 3D molecular understanding.
- We introduce a Cross-Modal Mask-Predict objective that enables the model to recover missing structural information from corrupted inputs, improving 3D modelling with multimodal signals. We also design a Reweighted Distance Prediction Loss to emphasize fine-grained distances and enhance spatial precision.
- Extensive experiments show that CrossMol achieves state-of-the-art performance on various molecular property prediction tasks. Ablation studies further confirm the effectiveness of our architecture.

### 1.1 Related works

Early methods for molecular pre-training primarily focused on 1D SMILES (Honda *et al.* 2019, Wang *et al.* 2019, Zheng *et al.* 2025a, 2025b) and 2D graph structures (Li *et al.* 2021, Lu *et al.* 2021, Xia *et al.* 2023). Recently, growing interest in 3D molecular data has emerged due to its richer representation of physical properties, including conformational details beyond 1D and 2D data. Given the multimodal nature of molecules, there is increasing research on joint modelling of multiple modalities to better capture semantic information. This section reviews recent advances in 3D and multimodal molecular pre-training.

### 1.2 3D molecular pre-training

Recent 3D molecular modelling methods use self-supervised learning on unlabelled data to extract informative features (Stärk *et al.* 2022, Liu *et al.* 2023, Zhou *et al.* 2023, Yu *et al.* 2024, Zheng *et al.* 2024). Most adopt encoder-only architectures, including Transformer-based models that encode atomic positions as attention biases (Luo *et al.* 2022, Zhou *et al.* 2023, Yu *et al.* 2024), and graph neural networks (GNNs) with message passing over edge features. Main objectives include geometry prediction (e.g. bond lengths, angles, and node types) (Fang *et al.* 2022, Luo *et al.* 2022, Zhou *et al.* 2023, Yu *et al.* 2024), coordinate denoising (Luo *et al.* 2022, Yu *et al.* 2024), and atomic distance prediction (Zhou *et al.* 2023, Yang *et al.* 2024), which enhance the model’s 3D understanding.

### 1.3 Multimodal molecular pre-training

Molecular data are inherently multimodal, each capturing different aspects of structure and properties, making effective integration a key challenge. Current approaches often treat modalities independently, aligning them via contrastive learning or cross-modal fusion (see Fig. 1). Contrastive learning methods use separate encoders to align representations across modalities (Liu *et al.* 2022a, 2023, Stärk *et al.* 2022), but struggle to jointly model them in downstream tasks. Zhu *et al.* (2022) integrate 2D and 3D data, yet their model still lacks support for mixed-modality input. Yu *et al.* (2024) propose randomly mixing modalities, retaining each with equal probability in the final input.

### 1.4 Cross-modal pre-training with CrossMol

In this section, we provide a detailed introduction to the cross-modal mask-predict pre-training model, denoted as CrossMol. This discussion encompasses the overall design philosophy, the details of the model architecture, and the training objectives.

### 1.5 Overview

To enhance the model’s ability to utilize multimodal information for 3D molecular understanding, we propose a modality fusion algorithm based on an Encoder-Decoder framework (Fig. 2). A Transformer-based SMILES Encoder first encodes the SMILES sequence, and its output is passed to a 3D Decoder via cross-attention to reconstruct corrupted 3D structures. This design enables each modality to be processed independently before being integrated in the 3D Decoder.

**Figure 2:**
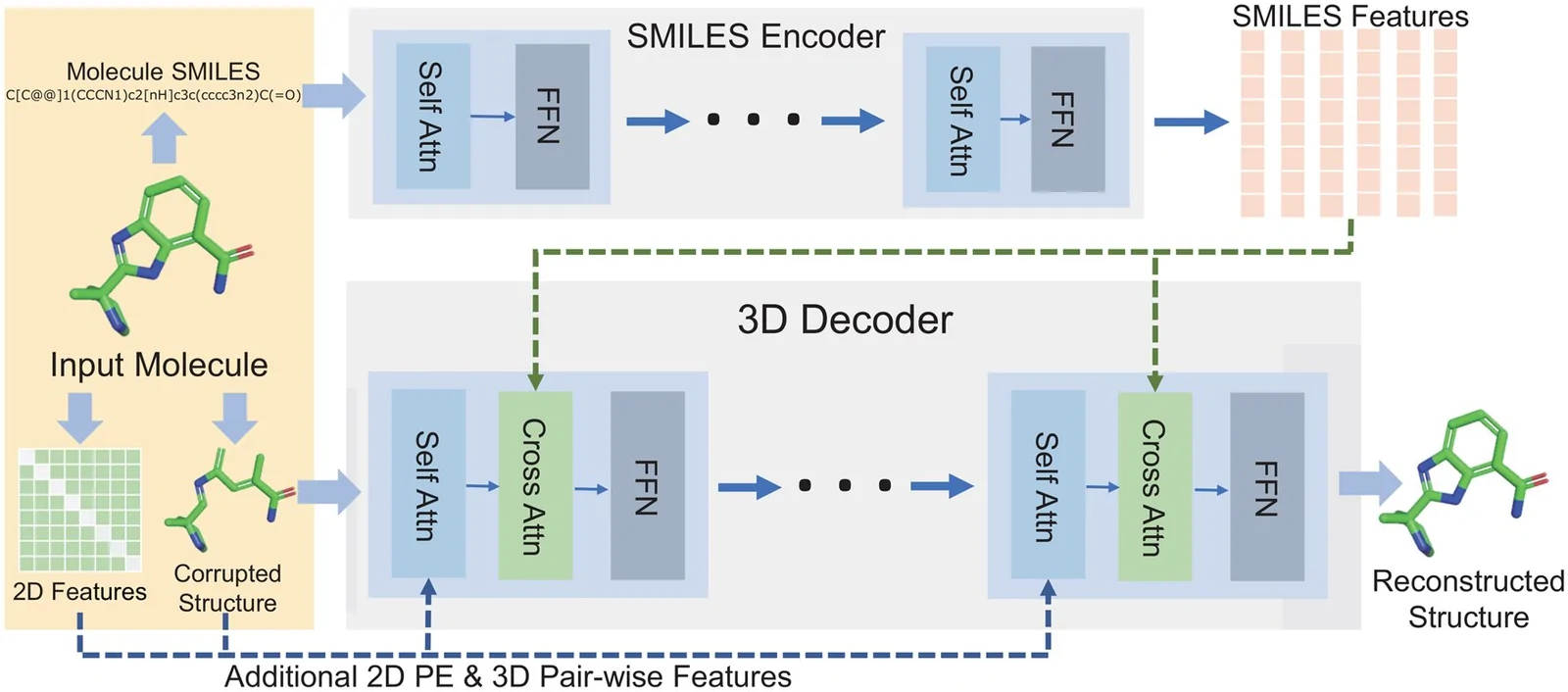
The overall framework of CrossMol. The SMILES Encoder takes SMILES information as input and extracts representations, which are then fed into the Decoder through a Cross-Attention mechanism. The Decoder’s task is to accurately predict the missing parts of the input 3D molecular data. In the diagram, Attn denotes attention, FFN denotes a feed-forward network, and PE denotes positional encoding.

To further improve 3D structural modelling, we introduce a Cross-Modal Mask-Predict Objective for self-supervised pre-training, where 2D information is incorporated as additional positional encoding to guide the prediction of missing 3D substructures. We also adopt a distance-aware reweighting strategy in the reconstruction loss to better capture fine-grained 3D details. Notably, the proposed cross-modal mask-predict paradigm is orthogonal to the choice of 3D backbone and can be readily applied to different geometry-aware architectures, including both SE(3)-equivariant models that explicitly model directional information and SE(3)-invariant approaches based on pair-wise distance encoding. Here SE(3) denotes the special Euclidean group in three dimensions. In this work, we adopt an SE(3)-invariant backbone similar to Uni-Mol (Zhou *et al.* 2023) for 3D structure modelling, while our asymmetric multimodal design is complementary to more expressive equivariant architectures.

### 1.6 Modality fusion with encoder-decoder architecture

The Encoder-Decoder architecture is crucial for achieving asymmetric modality fusion. Given the differences between 3D and SMILES information, we have designed a SMILES Encoder module to process SMILES input, and a 3D Decoder module to model the structural information. Additionally, we have incorporated the representations extracted by the SMILES Encoder into the cross-attention mechanism of the 3D Decoder, allowing the 3D Decoder to integrate information from different modalities. The following sections provide detailed descriptions of these model components.

### 1.7 SMILES encoder

The SMILES Encoder, represented as ${\text{Enc}}_{\theta }(X_{i}^{S})$, takes molecular SMILES information $X_{i}^{S}$ as input and produces the SMILES representation $S_{i}$ as output. Considering the similarity in format between SMILES input and textual data, we have opted to use the Transformer (Vaswani *et al.* 2017), a popular architecture in text modelling, as the foundational structure for the SMILES Encoder. Here are the details of Transformer in SMILES Encoder.

### 1.8 Transformer block

A Transformer is composed of a sequence of Transformer blocks, each containing a multi-head self-attention layer and a feed-forward layer. Let $X^{l}={\left[x_{1}^{l},x_{2}^{l},\ldots ,x_{n}^{l}\right]}^{⊤}\in R^{n\times d}$ represent the input to the *l*-th block, where each $x_{i}^{l}$ corresponds to the *l*-th representation of atom $a_{i}$. For any input $X^{l}$, the output $X^{l+1}$ is computed as follows:


$$\begin{array}{c} Q^{l,h}=X^{l}W_{Q}^{l,h},K^{l,h}=X^{l}W_{K}^{l,h},V^{l,h}=X^{l}W_{V}^{l,h}\\ {\text{Attention}}^{h}(X^{l})=\mathrm{softmax}\left(\frac{Q^{l,h}{\left(K^{l,h}\right)}^{⊤}}{\sqrt{d}}\right)V^{l,h}\\ {\tilde{X}}^{l}=\text{LN}(X^{l}+\sum_{h=1}^{H}{\;\text{Attention}}^{h}(X^{l})W_{O}^{l,h})\\ X^{l+1}=\text{LN}({\tilde{X}}^{l}+\text{GELU}({\tilde{X}}^{l}W_{1}^{l})W_{2}^{l}) \end{array}$$

where $W_{Q}^{l,h}$, $W_{K}^{l,h}$, $W_{V}^{l,h}$, $W_{O}^{l,h}$, $W_{1}^{l}$, and $W_{2}^{l}$ are learnable parameters, *h* is the number of attention heads, LN denotes layer normalization (Ba *et al.* 2016), and GELU denotes the Gaussian error linear unit.

### 1.9 3D decoder with 3D feature

In contrast to the SMILES Encoder, the 3D Decoder is designed to handle disrupted 3D structures and predict a complete 3D conformation. The standard Transformer cannot effectively model 3D data due to the continuous nature of coordinates and their non-invariance under global rotations and translations. To address this, following previous work (Luo *et al.* 2022), we encode Euclidean distances between atom pairs using a Gaussian Basis Kernel (Scholkopf *et al.* 1997), mapping the distance between atoms $a_{i}$ and $a_{j}$ to a pair feature vector $b^{ij}=[b_{1}^{ij},b_{2}^{ij},\ldots ,b_{H}^{ij}]$. We denote the pair feature in the *l*-th Transformer layer as $b^{ij,l}$, with $b^{ij,0}=b^{ij}$. To capture evolving pair-wise relationships, we update $b^{ij,l+1}$ during Transformer propagation:


$$b_{h}^{ij,l+1}=b^{ij,l}h+Q^{l,h}{\left(K^{l,h}\right)}^{⊤}$$

These pair-wise features also act as spatial positional encoding, helping the model distinguish between atoms. We incorporate them into the attention function of the decoder for 3D modelling:


$${\text{Attention}\;3D}^{h}(X^{l})=\text{softmax}\left(\frac{Q^{l,h}{\left(K^{l,h}\right)}^{⊤}}{\sqrt{d}}+b_{h}^{ij,l-1}\right)V^{l,h}$$

### 1.10 Cross-attention in decoder

To enable the 3D Decoder to integrate SMILES information, we have implemented a cross-attention mechanism that merges the output features from SMILES Encoder with the features in 3D Decoder. Here are the details of the cross-attention mechanism.

Let $S^{L}={\left[s_{1}^{L},s_{2}^{L},\ldots ,s_{n}^{L}\right]}^{⊤}\in R^{n\times d}$ represent the output of the final block in SMILES Encoder, where each $s_{i}^{L}$ corresponds to the *i*-th representation of SMILES token ${\text{SMILES}}_{i}$. The cross attention in decoder is computed as follows:


$$\begin{array}{c} \;Q^{l,h}=X^{l}W_{Q}^{l,h},K^{l,h}=S^{L}W_{K}^{l,h},V^{l,h}=S^{L}W_{V}^{l,h}\\ {\text{CrossAttn}}^{h}(X^{l},S^{L})=\mathrm{softmax}\left(\frac{Q^{l,h}{\left(K^{l,h}\right)}^{⊤}}{\sqrt{d}}\right)V^{l,h} \end{array}$$

where $W_{Q}^{l,h}$, $W_{K}^{l,h}$, and $W_{V}^{l,h}$ are learnable parameters, *h* is the number of attention heads.

### 1.11 Cross-modal mask-predict objective

To further enhance the model’s 3D modelling capability, we introduce the Cross-Modal Mask-Predict (Mask-P) Objective. Specifically, we drop parts of the molecular structure, input the corrupted structure into the model, and task it with predicting the original configuration. We employ a reweighted distance prediction loss to assign higher weights to more detailed distance information, facilitating the learning of meaningful 3D structural patterns through self-supervised training.

To help the model distinguish atoms in the dropped regions, we incorporate 2D Information $\Psi_{2D}$ as additional Positional Encoding (PE) in the 3D Decoder. This provides contextual cues about inter-atomic relationships and distances, improving 3D structure reconstruction.

The Cross-Modal Mask-Predict objective can be formalized as follows:


$$L_{\text{MaskP}}=\frac{1}{K}\sum_{i=1}^{K}L_{3D}(X_{i},f_{\theta }(D(X_{i}),{\text{SMILES}}_{i},\Psi_{2D}^{i}))$$

where D is the molecular substructure dropping module, $L_{3D}$ is the 3D structure reconstruction loss (atom type prediction, reweighted distance prediction, and coordinate prediction), $X_{i}$ represents the 3D structure of the *i*-th molecule, $\Psi_{2D}^{i}$ and ${\text{SMILES}}_{i}$ represents the 2D information and SMILES of the *i*-th molecule coordinates respectively. Here are more details of the objective.

### 1.12 Molecular substructure dropping

As highlighted in several studies (Wang *et al.* 2022a, Yang *et al.* 2024), employing a denoising objective can lead models to fit unreliable noisy distributions. Moreover, incorporating noisy structures into attention weights via 3D pair-wise features can disrupt the model’s attention mechanism. To address this, we perturb 3D structures by randomly removing a subset of atoms. Specifically, we select atoms at random, set their 3D pair-wise features $b^{ij}$ to zero, and replace their atomic types with the [MASK] symbol. This effectively removes both the structural and type information of these atoms from the model’s input. Such dropping is critical to train the model to recover missing atomic details in molecular structures.

### 1.13 Using 2D information as additional PE

In our model, 3D pair-wise features function as spatial positional encoding. However, during the substructure dropping phase, atomic structure and type information are removed, and their corresponding 3D pair-wise features are set to zero, making it difficult for the model to distinguish these atoms. To address this, we incorporate 2D structural features—such as shortest path distances and edge types—to provide additional positional cues.

Following Luo *et al.* (2022), for each atom pair (i,j), $\Psi_{\text{SPD}}^{ij}$ denotes their shortest path distance (SPD) in the molecular graph. Edge features along this path are aggregated into $\Psi_{\text{Edge}}^{ij}=\frac{1}{N}\sum_{n=1}^{N}w_{n}^{⊤}e_{n}$, where $e_{n}\in R^{d_{e}}$ are edge features and $w_{n}$ are learnable weights. The combined 2D feature $\Psi_{2D}=\Psi_{\text{SPD}}+\Psi_{\text{Edge}}$ is incorporated into the attention mechanism to help the model distinguish atoms even when 3D cues are missing.


$${\text{Attn}}_{3D\&2D}{\;}^{h}(X^{l})=\text{softmax}\left(\frac{Q^{l,h}{\left(K^{l,h}\right)}^{⊤}}{\sqrt{d}}+b_{h}^{ij,l-1}+\Psi_{2D}^{ij}\right)V^{l,h}$$

### 1.14 Reweighted distance prediction loss

Designing an appropriate structural reconstruction loss is crucial for 3D structure modelling. Existing frameworks typically assign equal training weights to distances across all scales, but long-range distances are more variable and unpredictable. For example, the distance between two substructures linked by a rotatable bond can fluctuate significantly. As a result, reconstruction loss is often dominated by such unstable long-range distances, causing the model to overlook important short-range information, as shown in Fig. 4. Short-range atomic interactions, such as bonded atoms, are generally stronger and essential for accurate modelling.

To address this, we propose a reweighted distance prediction (RDP) loss, as illustrated in Fig. 3. We reweight the loss based on distance scales to reduce the underemphasis on short-range information. The loss is defined as:


$$L_{\text{RDP}}(d,D)=\sum_{i,j}\frac{||d_{i,j}-D_{i,j}|{|}^{2}}{D_{i,j}}$$

where $d_{i,j}$ is the predicted distance between atoms *i* and *j*, and $D_{i,j}$ is the ground-truth distance.

**Figure 3:**
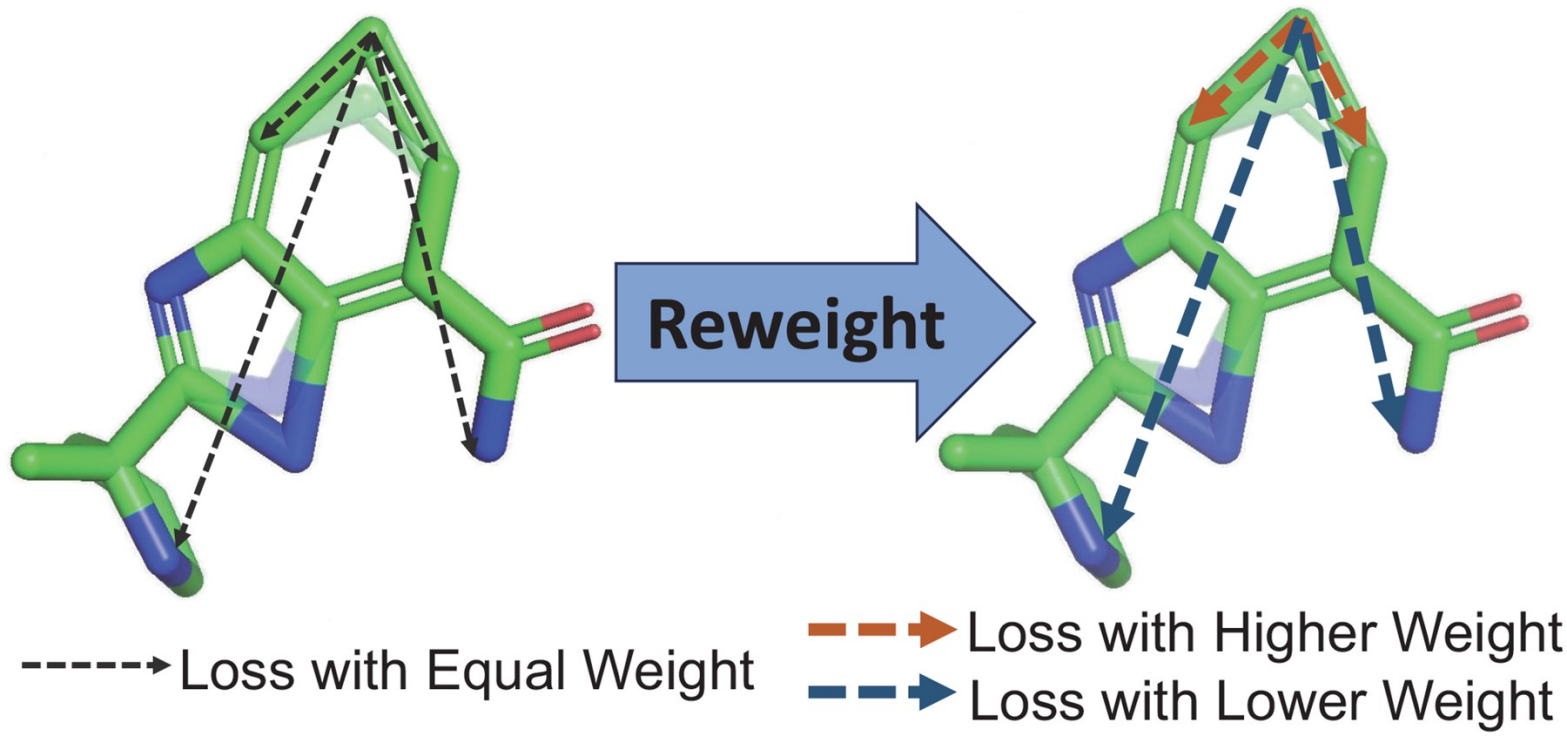
Reweighted distance prediction loss. Losses at different distance scales are assigned varying weights to ensure the model does not overlook some important short-range structural information, such as chemical bonds.

**Figure 4:**
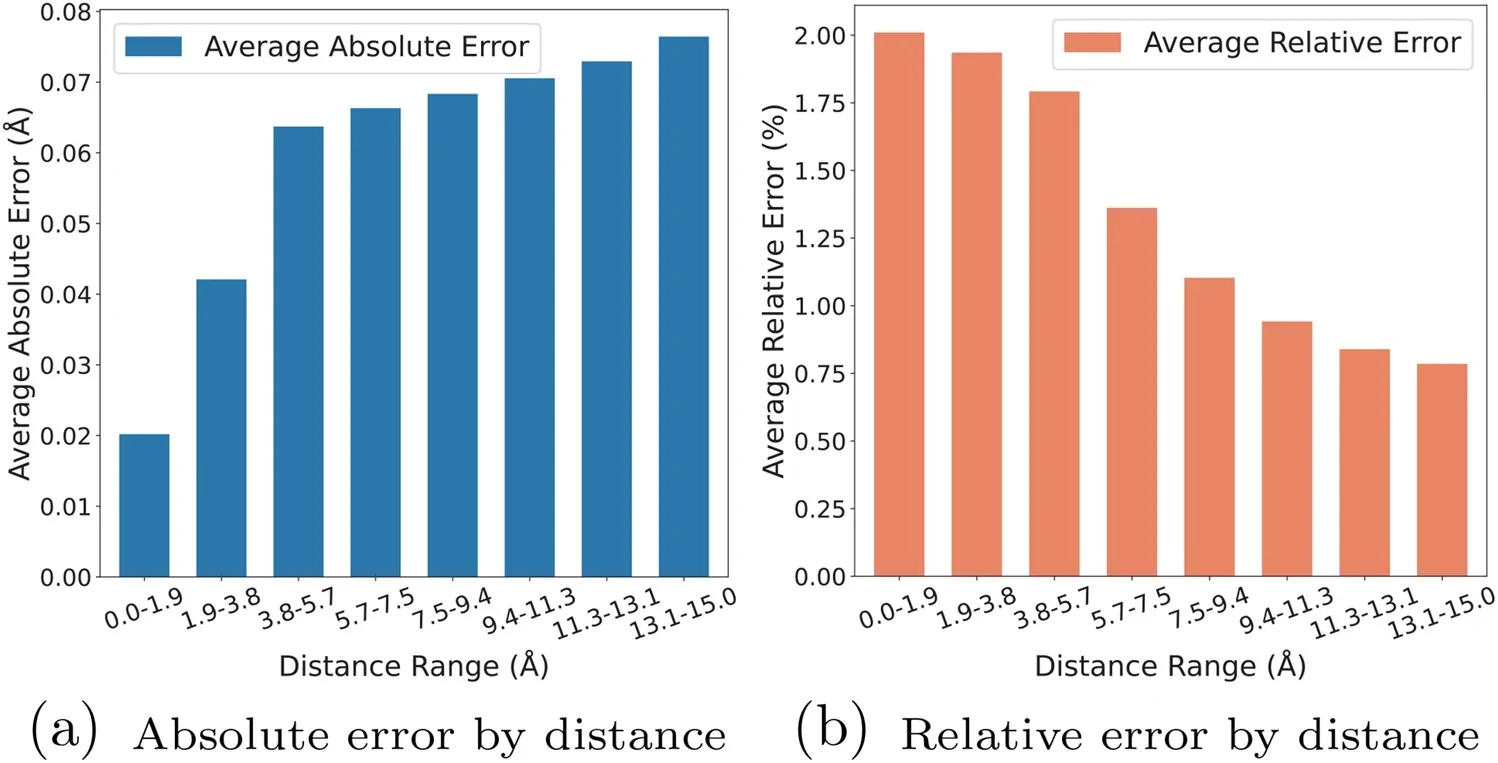
Structural reconstruction error across different distance scales. As shown in panel (a), the reconstruction error of the Uni-Mol rises as the distance scale increases, indicating that the structural reconstruction loss is dominated by long-range structural information. Panel (b) shows that Uni-Mol exhibits higher relative error in reconstructing short-range structural information. However, some important distance information, such as bond lengths (averaging around 1.4 Å), falls within this category.

## 2 Experiments

In this section, we present the experimental setup used in this paper and report the performance of the CrossMol model on molecular property prediction tasks. Additionally, we conduct analytical experiments to evaluate the effectiveness of the model’s design.

### 2.1 Experiment settings

#### 2.1.1 Datasets

For pre-training, we leverage the large-scale molecular dataset provided by Zhou *et al.* (2023), which includes 19 million molecules and 209 million conformations generated using experimental-torsion knowledge distance geometry (ETKDG) (Riniker and Landrum 2015) and the Merck Molecular Force Field (Halgren 1996). In this dataset, each molecule has 11 randomly generated conformations. For fine-tuning, we employ the widely recognized MoleculeNet benchmark (Wu *et al.* 2018), which comprises eight classification datasets and six regression datasets. We maintain the same data split as used in Zhou *et al.* (2023).

#### 2.1.2 Baselines

We evaluate our approach against a variety of supervised and pre-training baselines. The supervised methods include D-MPNN (Yang *et al.* 2019) and AttentiveFP (Xiong *et al.* 2020), both of which are based on GNNs. For pre-training, we consider several methods: N-gram (Liu *et al.* 2019), PretrainGNN (Hu *et al.* 2019), GROVER (Rong *et al.* 2020), GraphMVP (Liu *et al.* 2022a), MolCLR (Wang *et al.* 2022b), InfoGraph (Sun *et al.* 2019), Mole-BERT (Xia *et al.* 2023), MoleculeSDE (Liu *et al.* 2023), 3D InfoMax (Stärk *et al.* 2022), Uni-Mol (Zhou *et al.* 2023), and MoleBLEND (Yu *et al.* 2024). MoleBLEND currently represents the state-of-the-art on the benchmark.

#### 2.1.3 Implementation details

We use a Transformer with a hidden size of 512 and 64 attention heads, comprising six encoder layers and 15 decoder layers. During pre-training, the atom drop ratio is set to 0.15, and hydrogen atoms are excluded, consistent with all baselines. We use Adam ($\beta_{1}$ = 0.9, $\beta_{2}$ = 0.99) with a learning rate of 10^−4^, polynomial decay, 10,000 warm-up updates, a maximum of 1,000,000 updates, and a batch size of 128. For downstream evaluation, we follow the Uni-Mol fine-tuning protocol and compare against its official hydrogen-free checkpoint (Zhou *et al.* 2023). Since CrossMol and Uni-Mol are pre-trained on the same dataset, we keep the learning-rate schedule, warm-up strategy, batch size, and optimizer settings consistent. For molecular property prediction, CrossMol takes SMILES and 2D/3D structural information as input to leverage complementary multimodal signals.

#### 2.1.4 Main results

We evaluate CrossMol on the MoleculeNet (Wu *et al.* 2018) benchmark and compare its performance with baseline models. For the quantum chemistry (QM) tasks, which are heavily dependent on 3D structural knowledge, we provide only SMILES and 3D structural information to avoid interference from additional 2D data that could disrupt the model’s 3D modelling capability.

#### 2.1.5 Results on classification tasks

We evaluate CrossMol on eight widely used molecular property prediction tasks. The hyperparameters for each task are detailed in the supplementary materials. The area under the receiver operating characteristic curve (ROC-AUC) is used as the evaluation metric, and the results are summarized in Table 1. CrossMol achieves state-of-the-art (SOTA) performance on six tasks and closely matches the SOTA models on the remaining tasks. Additionally, CrossMol demonstrates the strongest average performance across all eight tasks, indicating that it outperforms other baseline models in these tasks.

**Table 1:** The overall results on eight molecular classification datasets.

Datasets	**BACE** ↑	**BBBP** ↑	**Tox21** ↑	**SIDER** ↑	**HIV** ↑	**MUV** ↑	**ClinTox** ↑	**ToxCast** ↑	**Mean** ↑
# Molecules	1531	2039	7831	1427	41,127	93,087	1478	8575	–
D-MPNN (Yang *et al.* 2019)	80.9(0.6)	71.0(0.3)	75.9(0.6)	57.0(0.7)	77.1(0.5)	78.6(1.4)	90.6(0.6)	65.5 (0.3)	74.6
Attentive FP (Xiong *et al.* 2020)	78.4(0.022)	64.3(1.8)	76.1(0.5)	60.6(3.2)	75.7(1.4)	76.6(1.5)	84.7(0.3)	63.7(0.2)	72.5
GROVER (Rong *et al.* 2020)	82.6(0.7)	70.0(0.1)	74.3(0.1)	64.8(0.6)	62.5(0.9)	67.3(1.8)	81.2(3.0)	65.4(0.4)	70.4
GraphMVP (Liu *et al.* 2022a)	81.2(0.9)	72.4(1.6)	75.9(0.5)	63.9(1.2)	77.0(1.2)	77.7(0.6)	79.1(2.8)	63.1(0.4)	73.8
InfoGraph (Sun *et al.* 2019)	77.8(0.8)	67.5(0.1)	73.2(0.4)	59.9(0.3)	75.1(0.9)	74.1(0.7)	76.5(1.0)	63.7(0.5)	71.0
MolCLR (Wang *et al.* 2022b)	82.4(0.9)	72.2(2.1)	75.0(0.2)	58.9(1.4)	78.1(0.5)	79.6(1.9)	91.2 (3.5)	69.2 (0.6)	70.8
Mole-BERT (Xia *et al.* 2023)	80.8(1.4)	71.9(1.6)	76.8(0.5)	62.8(1.1)	78.2(0.8)	78.6(1.8)	78.9(3.0)	64.3(0.2)	74.0
3D InfoMax (Stärk *et al.* 2022)	79.7(1.5)	69.1(1.0)	74.5(0.7)	60.6(0.7)	76.1(1.3)	74.4(0.7)	79.9(3.4)	64.4(0.8)	72.3
MoleculeSDE (Liu *et al.* 2023)	79.5(2.1)	71.8(0.7)	76.8(0.3)	60.8(0.3)	78.8(0.9)	**80.9** (0.3)	87.0(0.5)	65.0(0.2)	75.1
Uni-Mol (Zhou *et al.* 2023)	83.2(0.3)	71.5(0.2)	**78.9** (0.4)	57.7(1.8)	78.6(0.2)	72.6(0.8)	84.1(1.2)	69.1(0.1)	74.5
MoleBLEND (Yu *et al.* 2024)	83.7 (1.4)	73.0 (0.8)	77.8(0.8)	64.9 (0.3)	79.0 (0.8)	77.2(2.3)	87.6(0.7)	66.1(0.0)	76.2
Cross Mol	**86.3** (0.2)	**73.3** (0.3)	77.9 (0.6)	**66.4** (1.1)	**80.3** (0.5)	80.5 (1.6)	**93.7** (1.6)	**69.4** (0.2)	**78.8**

We report the area under the receiver operating characteristic curve (ROC-AUC; higher is better) under scaffold splitting. The best results are **bold**. The second-best results are underlined. Higher is better on all metrics.

#### 2.1.6 Results on regression tasks

We then evaluate the performance of CrossMol across 19 molecular regression tasks, using mean absolute error (MAE) and root mean square error (RMSE) as metrics. The comprehensive results across six datasets are summarized in Table 2. For datasets with multiple tasks, the mean MAE is calculated across all tasks. CrossMol outperforms other methods on five out of the six datasets, showcasing its effectiveness in regression tasks.

**Table 2:** The overall results on six molecular regression datasets under scaffold splitting.

Datasets	**QM9** ↓	**QM8** ↓	**QM7** ↓	**ESOL** ↓	**FreeSolv** ↓	**Lipo** ↓
# Molecules	133 885	21 789	6830	1129	642	4200
D-MPNN (Yang *et al.* 2019)	0.0081(0.00001)	0.0190(0.0001)	103.5(8.6)	1.050(0.008)	2.082(0.082)	0.683(0.016)
Attentive FP (Xiong *et al.* 2020)	0.0081(0.00001)	0.0179(0.001)	72.0(2.7)	0.877(0.029)	2.073(0.183)	0.721(0.001)
N-Gram ${\;}_{\text{RF}}$ (Liu *et al.* 2019)	0.0104(0.00016)	0.0236(0.0006)	92.8(4.0)	1.074(0.107)	2.688(0.085)	0.812(0.028)
PretrainGNN (Hu *et al.* 2019)	0.0092(0.00004)	0.0200(0.0001)	113.2(0.6)	1.100(0.006)	2.764(0.002)	0.739(0.003)
GROVER (Rong *et al.* 2020)	0.0099(0.00055)	0.0218(0.0004)	94.5(3.8)	0.983(0.090)	2.176(0.052)	0.817(0.008)
GraphMVP (Liu *et al.* 2022a)	–	–	–	1.029(0.033)	–	0.681(0.010)
MolCLR (Wang *et al.* 2022b)	–	0.0178(0.0003)	66.8(2.3)	1.271(0.040)	2.594(0.249)	0.691(0.004)
MoleBLEND (Yu *et al.* 2024)	–	–	–	0.831	1.910	0.638
Uni-Mol (Zhou *et al.* 2023)	0.0054 (0.0001)	0.0160 (0.0003)	58.9 (1.1)	0.844(0.022)	1.879 (0.011)	**0.610** (0.007)
Cross Mol	**0.005** (0.0001)	**0.0159** (0.0001)	**49.79** (1.2)	**0.820** (0.036)	**1.635** (0.021)	0.621 (0.006)

We report mean absolute error (MAE) on QM9, QM8, and QM7 tasks and root mean square error (RMSE) on ESOL, FreeSolv, and Lipo. The best results are **bold**. The second-best results are underlined. Lower is better on all metrics.

### 2.2 Analytical experiments

#### 2.2.1 Ablation studies on multimodal input

We conduct an ablation study on input modalities. As shown in Table 3, removing any modality significantly reduces performance, demonstrating that the model effectively leverages multimodal information. Removing 2D information (w/o 2D Info) leads to a larger drop than removing SMILES input (w/o SMILES), highlighting the strong contribution of 2D features. Further ablation on the two types of 2D features—SPD and Edge—shows that removing Edge information (w/o Edge) has a greater impact. This is consistent with Yu *et al.* (2024), and confirms the importance of bond type information in 2D molecular data. We also evaluate the effect of removing the SMILES encoder entirely (w/o SMILES Enc), where both the encoder and associated cross-attention modules are discarded. This variant performs similarly to the w/o SMILES setting, where the SMILES input is masked but the cross-attention structure is retained, with w/o SMILES slightly outperforming on average. These results highlight the importance of SMILES input, as removing it alone also leads to a noticeable performance drop, demonstrating its essential role in the model.

**Table 3:** Ablation studies.

Method	**BACE** ↑	**HIV** ↑	**SIDER** ↑	**ToxCast** ↑
w/o SMILES	84.8	78.4	63.5	69.1
w/o SMILES Enc	84.6	78.6	62.6	69.2
w/o 2D Info	71.2	79.3	61.2	66.1
w/o SPD	80.7	77.0	63.7	68.4
w/o Edge	71.2	77.5	62.7	67.5
w/o Reweight	82.7	77.9	60.9	68.8
w/o Mask-P	80.4	77.3	60.8	67.6
w/SMILES MLM	78.9	79.3	64.6	67.2
Cross Mol	**86.3**	**80.3**	**66.4**	**69.4**

We validate the effectiveness of multimodal inputs, model design, and training objectives. Best results are bold; second-best results are underlined.

w/o, without; w/, with; Enc, encoder; Info, information; SPD, shortest path distance; Mask-P, mask-predict; MLM, masked language model.

#### 2.2.2 Ablation studies on pre-training objectives

We perform ablation experiments on the cross-modal mask-predict objective (Mask-P Objective) and the reweighted distance prediction loss (RDP Loss) proposed in this work. As shown in Table 3, removing the RDP Loss (w/o Reweight) leads to a significant drop in model performance, indicating that the model’s ability to capture 3D structural information is compromised. Removing the Mask-P Objective (w/o Mask-P) also impacts performance, with a greater effect than removing the RDP Loss. This is because, without the Mask-P Objective, the model relies on traditional denoising for pre-training. The denoising process introduces noisy 3D Pair-wise Features, which degrade the model’s 3D structural modelling capability. These results confirm the effectiveness of the objectives.

#### 2.2.3 Ablation studies on quantum chemistry tasks

We report ablation results on three quantum chemistry tasks—QM9, QM8, and QM7—in Table 4. Unlike molecular property classification tasks, these tasks rely more on precise 3D structural modelling. Removing the Reweighted Distance Prediction Loss (w/o Reweight) leads to a larger performance drop than removing SMILES input or the SMILES encoder, highlighting its importance for capturing fine-grained 3D information. Adding a SMILES-side training objective (CrossMol w/SMILES MLM) results in the most severe performance degradation, suggesting it distracts the model from learning 3D structures. These results support the need for an asymmetric modality fusion strategy that leverages semantic cues from SMILES without compromising 3D learning.

**Table 4:** Ablation studies on QM tasks.

Method	**QM9** ↓	**QM8** ↓	**QM7** ↓
w/o SMILES	0.0052	0.0164	57.14
w/o SMILES Enc	0.0051	0.0162	58.37
w/o Reweight	0.0053	0.0165	59.49
w/SMILES MLM	0.0063	0.0181	63.67
Cross Mol	**0.0050**	**0.0159**	**49.79**

We validate the effectiveness of the model design on three quantum chemistry (QM) tasks. Best results are bold; second-best results are underlined. Abbreviations: w/o, without; w/, with; Enc, encoder; MLM, masked language model.

#### 2.2.4 Asymmetric multimodal models excel at structural modelling

To validate the effectiveness of the asymmetric design, we add a Masked Language Model (MLM) loss to the SMILES Encoder, allowing the model to receive both 1D and 3D training signals. This makes the two modalities more symmetric, but as shown in Table 3, it leads to performance drops on several tasks.

To investigate this, we compare structure reconstruction performance before and after adding SMILES MLM. As shown in Fig. 5, adding SMILES MLM increases the 3D atomic type prediction loss and hinders convergence, with validation loss failing to stabilize. This indicates that incorporating SMILES training weakens the model’s focus on 3D structure, impairing downstream performance. Therefore, focusing on 3D pre-training is crucial to strengthen 3D modelling capability, confirming the effectiveness of our asymmetric multimodal design.

**Figure 5:**
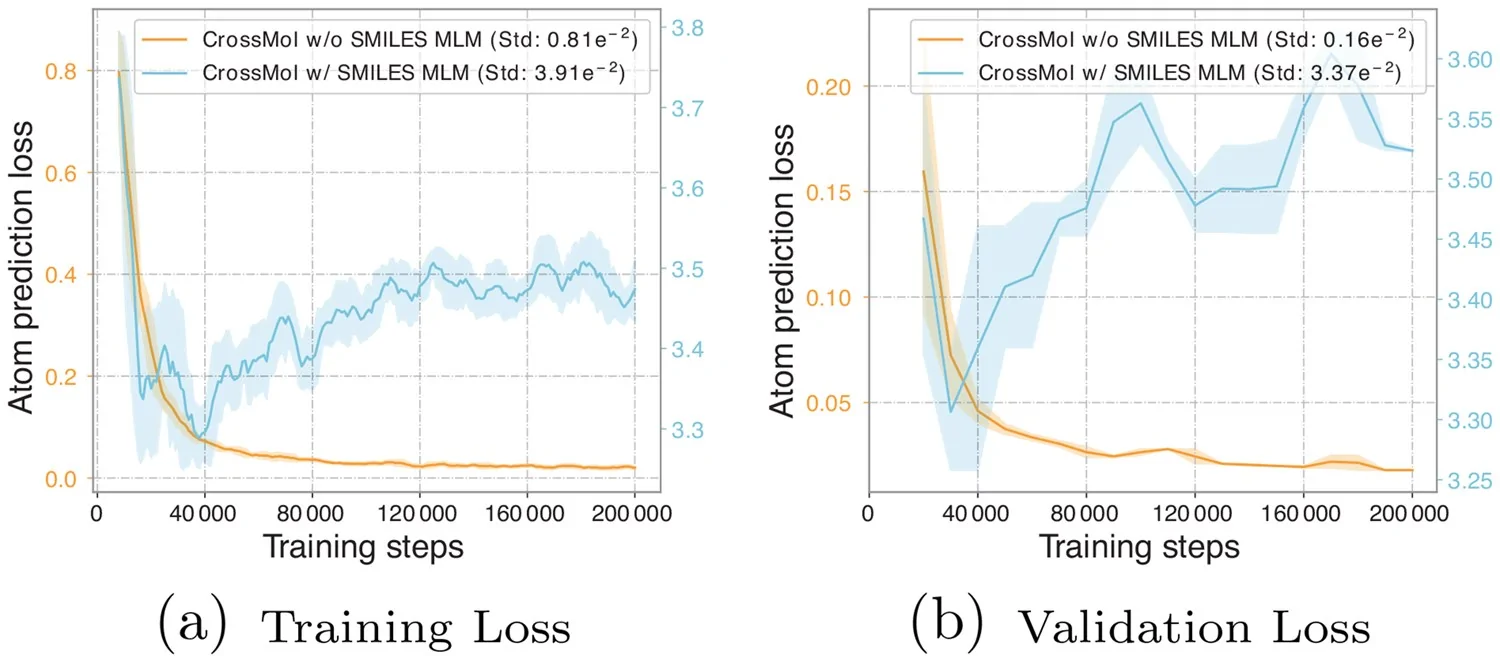
The loss curves with and without SMILES masked language model (MLM) training. CrossMol with SMILES MLM loss exhibits larger reconstruction loss values and fluctuations. The results demonstrate that adding SMILES training signal to the model causes CrossMol to overlook 3D structural information. In the legends, w/o means without, w/means with, and Std denotes standard deviation.

#### 2.2.5 2D Structural information enhances training stability

We compared the loss curves of the CrossMol model with and without Additional 2D PE during training. For the training loss, as shown in Fig. 6a, the model’s training loss exhibits significantly greater fluctuations without Additional 2D PE, with the standard deviation of these fluctuations being 10 times larger than when Additional 2D PE is provided. This indicates that including 2D PE enhances training stability.

**Figure 6:**
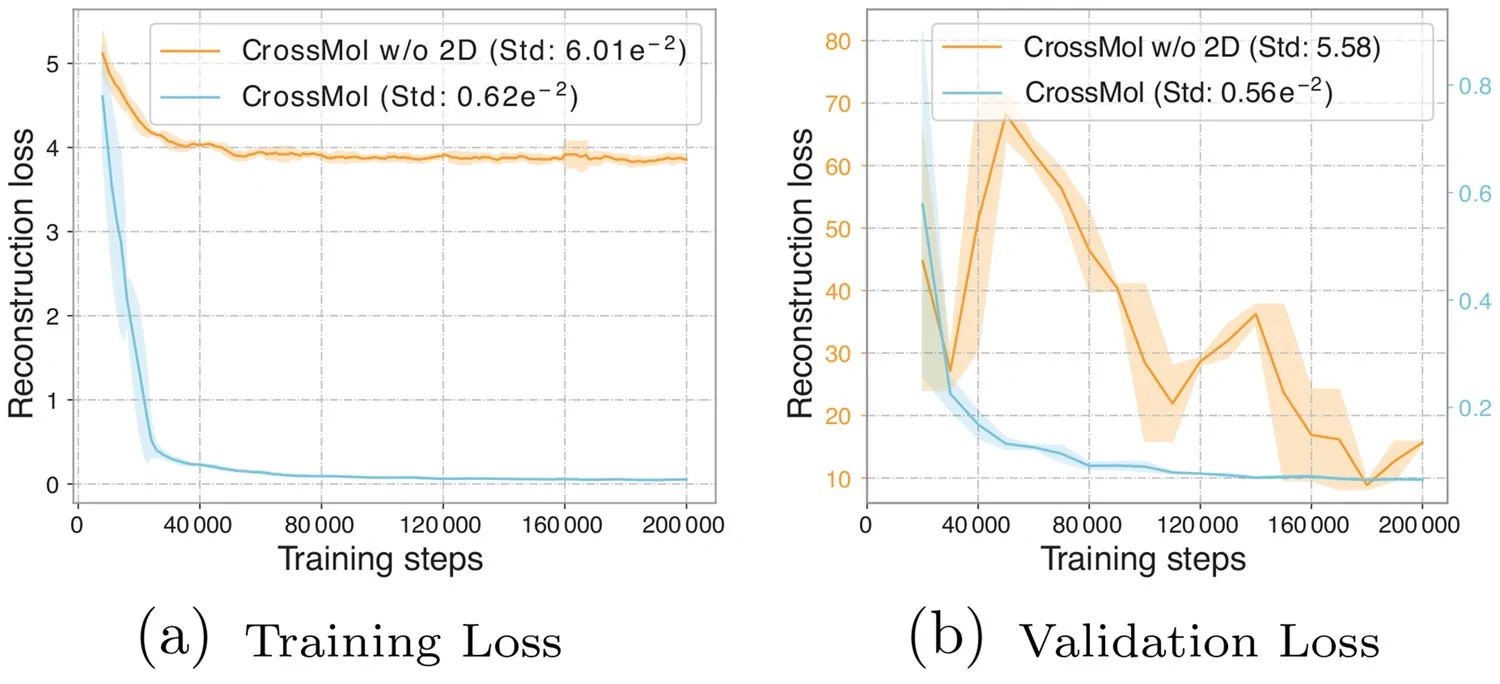
The loss curves with and without additional 2D positional encoding (PE). CrossMol w/o 2D information exhibits larger reconstruction loss values and fluctuations. The results demonstrate that providing Additional 2D PE enhances training stability. In the legends, w/o means without, and Std denotes standard deviation.

Further analysis of the validation loss curves, as shown in Fig. 6b, reveals that without Additional 2D PE, the validation loss is highly unstable and almost fails to converge, with fluctuations around 100 times greater than when Additional 2D PE is used. This suggests that without Additional 2D PE, the model struggles to complete the reconstruction task, further confirming the effectiveness of our 2D PE design.

#### 2.2.6 Multimodal inputs improve the quality of molecular representations

To further examine how multimodal information affects representation learning, we visualize molecular embeddings learned by CrossMol on the BACE dataset (Wu *et al.* 2018) with and without 1D and 2D inputs. Specifically, we apply t-distributed stochastic neighbour embedding (t-SNE) to project the learned representations into a two-dimensional space and compare the full multimodal model with its ablated variant that removes 1D and 2D information. As shown in Fig. 7, incorporating 1D and 2D inputs yields more structured and separable embeddings between classes, whereas removing these modalities produces more entangled representations with heavier class overlap. These results indicate that multimodal inputs provide complementary chemical and topological cues that improve the quality of learned molecular representations, supporting the effectiveness of CrossMol’s multimodal design.

**Figure 7:**
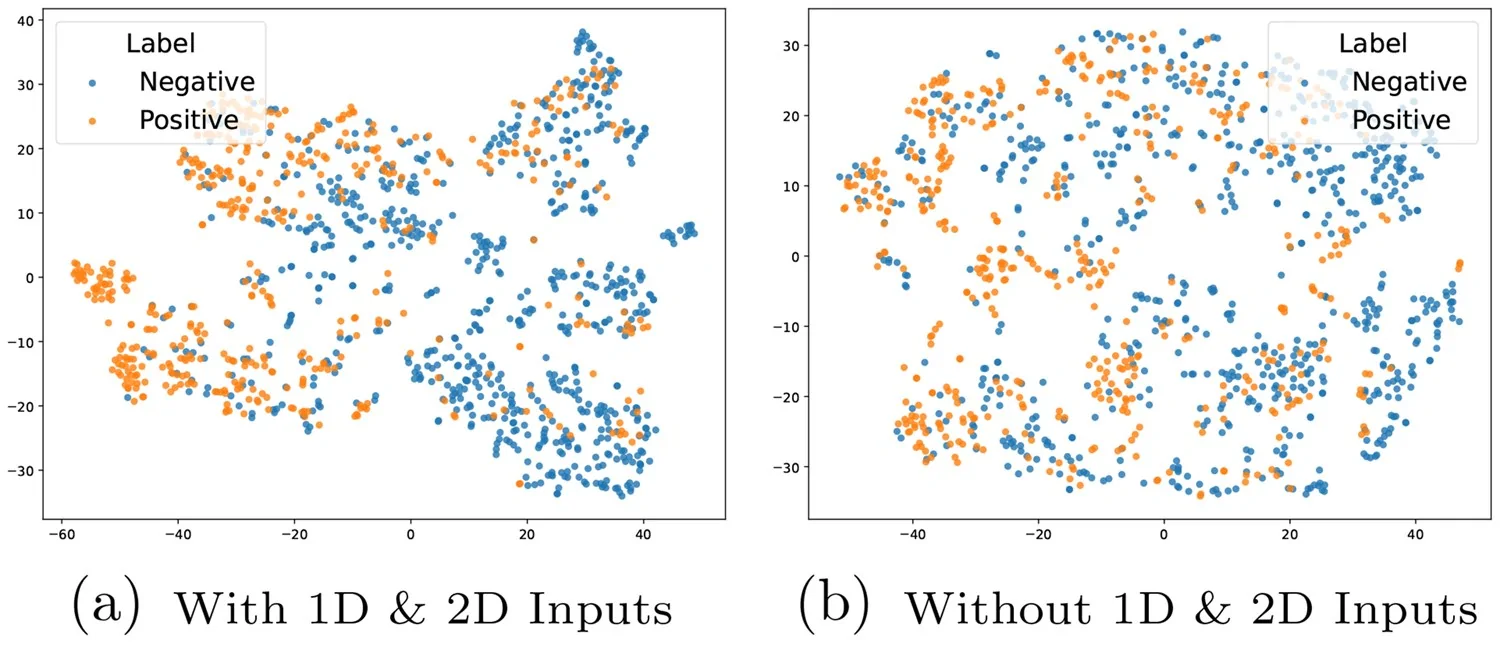
The embedding distributions with and without 1D and 2D inputs. CrossMol trained with 1D and 2D inputs produces more discriminative and structured representations, whereas removing 1D and 2D information leads to more entangled embeddings. The results demonstrate that multimodal inputs significantly improve the quality of learned molecular representations.

### 2.3 Computational efficiency

We further analyse the computational cost of CrossMol in comparison with Uni-Mol. Due to the introduction of the SMILES encoder and cross-attention modules, CrossMol has a larger model size (76.9 million versus 47.5 million parameters). In practice, CrossMol takes 95.6 h for pre-training on a single Tesla A100 graphics processing unit (GPU), compared to 36.2 h for Uni-Mol, with averaged GPU memory usage of 21.7 GB (vs. 18.4 GB for Uni-Mol). The inference latency is also higher (9.3 ms vs. 3.9 ms per sample).

Importantly, the overall training cost remains practical, as the model can be trained on a single A100 GPU. Given the substantial performance gains, we consider this trade-off between efficiency and predictive accuracy to be acceptable.

## 3 Conclusions

In this paper, we introduce a novel asymmetric multimodal molecular model, CrossMol. By incorporating SMILES information and introducing cross-modal mask-predict objective, CrossMol enhances the modelling ability of 3D molecular structures. We also prove that when using cross-modal mask-predict objective, adding 2D information to the model can improve the training stability. Extensive experiments on downstream tasks demonstrate that CrossMol achieves state-of-the-art performance in multiple molecular property prediction tasks. Additionally, numerous analytical experiments confirm the effectiveness of multimodal inputs, pre-training objectives, and the asymmetric design.

## Supplementary Material

btag702_Supplementary_Data

## Data Availability

The source code and training data for CrossMol are publicly available at https://github.com/zhengkangjie/crossmol. The code is archived with DOI: https://doi.org/10.5281/zenodo.19653204.
