# Supplementary material for: Cross Mol: cross-modal mask-predict pre-training for 3D molecular data
Source: Bioinformatics. 2026 Sep 23;42(10):btag702. doi: 10.1093/bioinformatics/btag702 (PMC13645429; doi:10.1093/bioinformatics/btag702)
Supplement: Supplementary file 1 [file btag702_supplementary_data.pdf]

Supplementary Material

Datasets

Pre-training Datasets We use the dataset provided by Zhou et al. (2023), which contains 19M molecules and 209M conformations generated by ETKGD (Riniker and Landrum 2015) and Merck Molecular Force Field (Halgren 1996). During the pre-training process, to ensure training efficiency, we remove all hydrogen atoms in the pre-training dataset.

Fine-tuning Datasets We conduct experiments on the MoleculeNet(Wu et al. 2018) benchmark in the molecular property prediction task. MoleculeNet serves as a widely recognized benchmark in the field of molecular property prediction. Here, we offer the statistics and basic information of the MoleculeNet benchmark datasets in Table 1.

Table 1: Summary information of the MoleculeNet benchmark datasets.

Dataset	Tasks	Task type	Molecules (train/valid/test)	Describe
QM7	1	Regression	5,464/685/681	Electronic properties
QM8	12	Regression	17,428/2,179/2,179	Excited state properties
QM9	3	Regression	107,108/13,388/13,388	Energetic, electronic and thermodynamic properties
ESOL	1	Regression	902/113/113	Water solubility
FreeSolv	1	Regression	513/64/64	Hydrogen free energy
Lipo	1	Regression	3,360/420/420	Octanol/water distribution ratio, coefficient
BACE	1	Classification	1,210/151/151	Binding results of human BACE-1 inhibitors
BBBP	1	Classification	1,631/204/204	Blood-brain barrier penetration
ClinTox	2	Multi-label classification	1,182/148/148	Clinical trial toxicity and FDA approval status
Tox21	12	Multi-label classification	6,264/783/783	Qualitative toxicity measurements
ToxCast	617	Multi-label classification	6,860/858/858	Toxicology data based on in vitro screening
SIDER	27	Multi-label classification	1,141/143/143	Adverse drug reactions to the 27 systemic organs
HIV	1	Classification	32,901/4,113/4,113	The ability to suppress HIV replication
MUV	17	Multi-label classification	74,469/9,309/9,309	A subset of PubChem BioAssay

Evaluation protocol of QM9. QM9 contains several quantum mechanical properties of different quantitative ranges, and we select *homo, lumo and gap* of similar quantitative range, following the setup of the previous work (Zhou et al. 2023).

Hyper-Parameter Configuration

We implement CROSSMOL using 6 stacked Transformer layers in encoder and 15 stacked Transformer layers in decoder, each with 64 attention heads. The model dimension and feedforward dimension of each Transformer layer are 512 and 2048. The total number of CROSSMOL's parameters is 76M. We use Adam (Kingma and Ba 2014) and polynomial learning rate scheduler to train CROSSMOL and set the learning rate 1e-4, weight decay 1e-4, warmup step 10K. The total training step is 1M and each batch has 128 samples at maximum. We train CROSSMOL on a single NVIDIA A100 GPU for about 2 days.

For more pre-training hyper-parameters, please refer to Table 2.

In downstream tasks, we adopted the same hyper-parameters as Uni-Mol(Zhou et al. 2023) during pre-training, and performed a similar hyper-parameter search during downstream fine-tuning to find the optimal settings. The search space is shown in Table 3.

In different downstream task, we use different hyper-parameters. We ran CROSSMOL three times with different random seeds for each task and reported the mean and standard deviation of the results For detailed fine-tuning hyper-parameters, please refer to Table 4.

Full Results on QM9

Our evaluation on the QM9 dataset follows the same protocol as Uni-Mol and GEM, where three tasks with similar quantitative ranges—HOMO, LUMO, and Gap—are selected, and the average prediction error across these tasks is used as the final metric. We also agree with your point, and therefore also report CROSSMOL's performance on each of the three tasks individually. As shown in Table 5, CROSSMOL outperforms Uni-Mol on all three tasks. However, since CROSSMOL uses the same pre-training data as Uni-Mol—where molecular conformations are generated using ETKGD with Merck Molecular Force Field optimization—the conformations are of lower quality compared to those in MoleBlend(Yu et al. 2024), which uses DFT-optimized structures from the PCQM4Mv2 dataset. In addition, CROSSMOL does not use any supervised labels (e.g., conformation potentials) during pre-training. These factors contribute to the performance gap between CROSSMOL and MoleBlend(Yu et al. 2024) on such tasks.

Table 2: CROSSMOL hyper-parameters for pre-training.

Hyper-parameters	Value
Learning rate	1e-4
LR scheduler	polynomial_decay
Warmup updates	10K
Max updates	1M
Batch size	128
Reweighted Distance loss function and its weight	Smooth L1, 10.0
Coordinate loss function and its weight	Smooth L1, 5.0
Atom loss function and its weight	Cross entropy, 1.0
FFN dropout	0.1
Attention dropout	0.1
Embedding dropout	0.1
Num of encoder layers	6
Num of encoder attention heads	64
Encoder embedding dim	512
Encoder FFN dim	2048
Num of decoder layers	15
Num of decoder attention heads	64
Decoder embedding dim	512
Decoder FFN dim	2048
Adam (β_1, β_2)	(0.9,0.99)
Drop ratio	0.15
Vocabulary size (atom types)	30
Activation function	GELU

Table 3: Search space for downstream tasks: [...] denotes a continuous interval, and {...} denotes discrete values. Small datasets include BBBP, BACE, ClinTox, Tox21, ToxCast, SIDER, ESOL, FreeSolv, Lipo, QM7, and QM8; large datasets include MUV and QM9.

**Hyperparameter**	**Small**	**Large**	**HIV**
Learning rate	[1e^{-5}, 5e^{-4}]	{2e^{-5}, 1e^{-4}}	{2e^{-5}, 5e^{-5}}
Batch size	{32, 64, 128, 256}	{128, 256}	{128, 256}
Epochs	[20, 200]	{20, 40}	{2, 5, 10}
Pooler dropout	{0.0, 0.1, 0.2, 0.5}	{0.0, 0.1}	{0.0, 0.2}
Warmup ratio	[0.0, 0.4]	{0.0, 0.06}	{0.0, 0.1}

Full Ablation Results

The full ablation results on all eight classification tasks are now provided in Table 6. The original conclusions still remain valid, confirming the effectiveness of multimodal inputs, training objectives, and model design.

Validation for Cross-modal Alignment

We have evaluated CROSSMOL on the molecular conformation generation task. Specifically, this task aims to predict a more accurate 3D conformation of a molecule given its SMILES and a noisy initial 3D structure.

We followed the same experimental setup as Uni-Mol(Zhou et al. 2023), using the GEOM-QM9 and GEOM-Drugs datasets, and adopted Coverage (COV) and Matching (MAT) as evaluation metrics. The results are presented in Table 7. As shown, CROSSMOL outperforms the baseline models, particularly Uni-Mol. Since CROSSMOL and Uni-Mol share the same training data and task setup, the performance improvement demonstrates that CROSSMOL benefits from multimodal information fusion, leading to stronger alignment across modalities in this task.

Comparison with UniCorn

While UniCorn(Feng et al. 2024) does report stronger performance than CROSSMOL on several tasks, it uses a different pre-training dataset and model backbone. In our earlier experiments, to ensure fair comparison, CROSSMOL was pre-trained using exactly the same data and settings as Uni-Mol. Despite this, CROSSMOL consistently outperformed Uni-Mol across nearly all tasks, demonstrating the advantage of the proposed multimodal framework. Furthermore, when we replaced CROSSMOL's SMILES encoder with a pre-trained SMILES encoder, the performance improved across multiple MoleculeNet tasks and even surpassed UniCorn (as shown in Table 8), indicating that CROSSMOL still has room for further improvement.

Table 4: CrossMol hyper-parameters for fine-tuning.

Tasks	Epochs	Batch size	Learning rate	Warmup Ratio	Dropout	Pooler-dropout
BACE	20	64	1e-4	0.36	0.1	0.2
BBBP	40	128	4e-4	0.18	0.1	0.1
TOX21	80	128	1e-4	0.06	0.1	0.1
SIDER	40	32	5e-4	0.5	0.1	0
HIV	5	256	5e-5	0.1	0.1	0.2
MUV	20	128	2e-5	0.3	0.1	0.1
ClinTox	80	256	5e-5	0.25	0.1	0.7
ToxCast	160	64	1e-4	0.06	0.1	0.2
QM9	40	128	1e-4	0.06	0.1	0
QM8	120	32	1e-4	0.02	0	0
QM7	200	32	3e-4	0.06	0.1	0.1
ESOL	200	256	5e-4	0.06	0.1	0.4
FreeSolv	160	64	8e-5	0.1	0.1	0.4
Lipo	100	64	1e-4	0.24	0.1	0.1

Table 5: Task-wise Performances of CrossMol and Uni-Mol on QM9.

Method	HOMO ↓	LUMO ↓	Gap↓	MAE↓
Uni-Mol	0.0040	0.0055	0.0066	0.0054
CrossMol	**0.0039**	**0.0050**	**0.0062**	**0.0050**

Pre-training Loss Curves of Ablation Studies

To examine the contributions of 1D information, 2D information, and the pre-training objectives in the CrossMol model, we plot the training curves for the corresponding ablation studies in Figures 1. It is worth noting that, to better reflect the overall training dynamics of the model, these training curves report the total loss during training, which corresponds to the Cross-Modal Mask-Predict Objective in the original paper.

In Figure 1(a), we show the training curves for the ablations related to 1D information. We observe that removing the SMILES input (CrossMol w/o SMILES) results in a noticeable increase in loss during the later stages of training. This is because the model struggles more to reconstruct the missing 3D structures without atom type and bond information provided by the SMILES input. On the other hand, introducing an additional SMILES-side training signal (CrossMol w/ SMILES MLM) shifts the model's focus away from 3D structure modeling, leading to a significant increase in both the overall training loss and its variance.

In Figure 1(b), we present the training curves from the 2D information ablation study. The results show that removing 2D information (CrossMol w/o 2D Info) leads to a significant increase in loss, indicating the importance of topological cues (e.g., positional encodings) for accurate 3D reconstruction. Moreover, removing chemical bond information (CrossMol w/o Edge) has a more adverse impact on model stability than removing shortest path distances (CrossMol w/o SPD).

In Figure 1(C), we report the training curves from the pre-training objective ablation. Removing the Mask-Predict objective (CrossMol w/o Mask-P) and instead training the model using a standard denoising approach on 3D structure data simplifies the learning task, which results in a lower loss overall.

In summary, these ablation results demonstrate that leveraging the direct and topological insights from 1D and 2D data—such as atom types, chemical bonds, and molecular graph structure—is beneficial for enhancing the model's ability to understand and reconstruct fine-grained 3D molecular features.

Performance on Few-shot Settings

To evaluate the model's robustness in low-resource settings, we conducted additional experiments under few-shot scenarios using 10% and 20% of the training data. As shown in Table 9, CrossMol outperforms Uni-Mol across multiple datasets, particularly on HIV, SIDER, and ToxCast. These results demonstrate that CrossMol is more data-efficient and highlight the benefits of incorporating multimodal information, especially when training data is limited.

Table 6: Full Ablation Results of CrossMol.

Method	BACE ↑	BBBP ↑	Tox21 ↑	SIDER ↑	HIV ↑	MUV ↑	ClinTox ↑	ToxCast ↑	Mean ↑
w/o SMILES	<u>84.8</u>	69.7	**78.4**	63.5	78.4	**81.0**	71.7	69.0	74.6
w/o SMILES Enc	84.6	71.5	78.0	62.6	78.6	79.5	71.3	<u>69.2</u>	74.3
w/o 2D Info	71.2	72.3	76.6	61.2	<u>79.3</u>	72.1	90.9	66.1	73.7
w/o SPD	80.7	<u>72.9</u>	77.1	63.7	77.0	74.0	92.7	68.4	75.8
w/o Edge	71.2	69.8	77.8	62.7	77.5	73.1	91.8	67.5	73.9
w/o Reweight	82.7	71.8	<u>78.1</u>	60.9	77.9	75.4	92.0	68.8	<u>76.0</u>
w/o Mask-P	80.4	70.9	76.3	60.8	77.3	73.4	93.1	67.6	75.0
w/ SMILES MLM	78.9	70.9	77.8	<u>64.6</u>	<u>79.3</u>	65	**94.8**	67.2	74.8
CrossMol	**86.3**	**73.3**	77.9	**66.4**	**80.3**	<u>80.5</u>	<u>93.7</u>	**69.4**	**78.8**

Table 7: Performance on Molecular Conformation Generation Tasks.

Methods	QM9 COV(↑, %)		QM9 MAT(↓, Å)		Drugs COV(↑, %)		Drugs MAT(↓, Å)	
	Mean	Median	Mean	Median	Mean	Median	Mean	Median
RDKit	83.26	90.78	0.3447	0.2935	60.91	65.70	1.2026	1.1252
CVGAE	0.09	0.00	1.6713	1.6088	0.00	0.00	3.0702	2.9937
GraphDG	73.33	84.21	0.4245	0.3973	8.27	0.00	1.9722	1.9845
CGCF	78.05	82.48	0.4219	0.3900	53.96	57.06	1.2487	1.2247
ConfVAE	80.42	85.31	0.4066	0.3891	53.14	53.98	1.2392	1.2447
ConfGF	88.49	94.13	0.2673	0.2685	62.15	70.93	1.1629	1.1596
GeoMol	71.26	72.00	0.3731	0.3731	67.16	71.71	1.0875	1.0586
DGSM	91.49	95.92	0.2139	0.2137	78.73	94.39	1.0154	0.9980
GeoDiff	92.65	95.75	0.2016	0.2006	88.45	97.09	0.8651	0.8598
DMCG	94.98	98.47	0.2365	0.2312	91.27	100.00	0.8287	0.7908
UniMol	<u>97.95</u>	<u>100.00</u>	<u>0.1831</u>	<u>0.1659</u>	<u>91.91</u>	<u>100.00</u>	<u>0.7863</u>	<u>0.7794</u>
CrossMol	**98.66**	**100.00**	**0.1735**	**0.1613**	**92.75**	**100.00**	**0.6753**	**0.7076**

Table 8: The overall results on 8 molecule classification datasets. The best results are **bold**. The second-best results are <u>underlined</u>. Higher is better on all metrics.

Datasets<br># Molecules	BACE↑<br>1531	BBBP↑<br>2039	Tox21↑<br>7831	SIDER↑<br>1427	HIV↑<br>41127	MUV↑<br>93087	ClinTox↑<br>1478	ToxCast↑<br>8575	Mean↑<br>-
D-MPNN	80.9(0.6)	71.0(0.3)	75.9(0.6)	57.0(0.7)	77.1(0.5)	78.6(1.4)	90.6(0.6)	65.5(0.3)	74.6
Attentive FP	78.4(0.022)	64.3(1.8)	76.1(0.5)	60.6(3.2)	75.7(1.4)	76.6(1.5)	84.7(0.3)	63.7(0.2)	72.5
GROVER	82.6(0.7)	70.0(0.1)	74.3(0.1)	64.8(0.6)	62.5(0.9)	67.3(1.8)	81.2(3.0)	65.4(0.4)	70.4
GraphMVP	81.2(0.9)	72.4(1.6)	75.9(0.5)	63.9(1.2)	77.0(1.2)	77.7(0.6)	79.1(2.8)	63.1(0.4)	73.8
InfoGraph	77.8(0.8)	67.5(0.1)	73.2(0.4)	59.9(0.3)	75.1(0.9)	74.1(0.7)	76.5(1.0)	63.7(0.5)	71.0
MolCLR	82.4(0.9)	72.2(2.1)	75.0(0.2)	58.9(1.4)	78.1(0.5)	79.6(1.9)	91.2(3.5)	69.2(0.6)	75.8
Mole-BERT	80.8(1.4)	71.9(1.6)	76.8(0.5)	62.8(1.1)	78.2(0.8)	78.6(1.8)	78.9(3.0)	64.3(0.2)	74.0
3D InfoMax	79.7(1.5)	69.1(1.0)	74.5(0.7)	60.6(0.7)	76.1(1.3)	74.4(0.7)	79.9(3.4)	64.4(0.8)	72.3
MoleculeSDE	79.5(2.1)	71.8(0.7)	76.8(0.3)	60.8(0.3)	78.8(0.9)	80.9(0.3)	87.0(0.5)	65.0(0.2)	75.1
Uni-Mol	83.2(0.3)	71.5(0.2)	<u>78.9</u>(0.4)	57.7(1.8)	78.6(0.2)	72.6(0.8)	84.1(1.2)	69.1(0.1)	74.5
MoleBLEND	83.7(1.4)	73.0(0.8)	77.8(0.8)	64.9(0.3)	79.0(0.8)	77.2(2.3)	87.6(0.7)	66.1(0.0)	76.2
UniCorn	<u>85.8</u>(1.2)	<u>74.2</u>(1.1)	**79.3**(0.5)	64.0(1.8)	79.8(0.9)	<u>82.6</u>(1.0)	92.1(0.4)	69.4(1.1)	78.4
CrossMol	**86.3**(0.2)	73.3(0.3)	77.9(0.6)	**66.4**(1.1)	<u>80.3</u>(0.5)	80.5(1.6)	<u>93.7</u>(1.6)	<u>69.4</u>(0.2)	<u>78.8</u>
CrossMol (Pre-trained Enc)	84.7(0.6)	**74.5**(0.5)	78.3(0.5)	<u>66.1</u>(0.8)	**83.0**(0.9)	**82.8**(1.4)	**96.2**(2.1)	**71.7**(0.7)	**79.7**

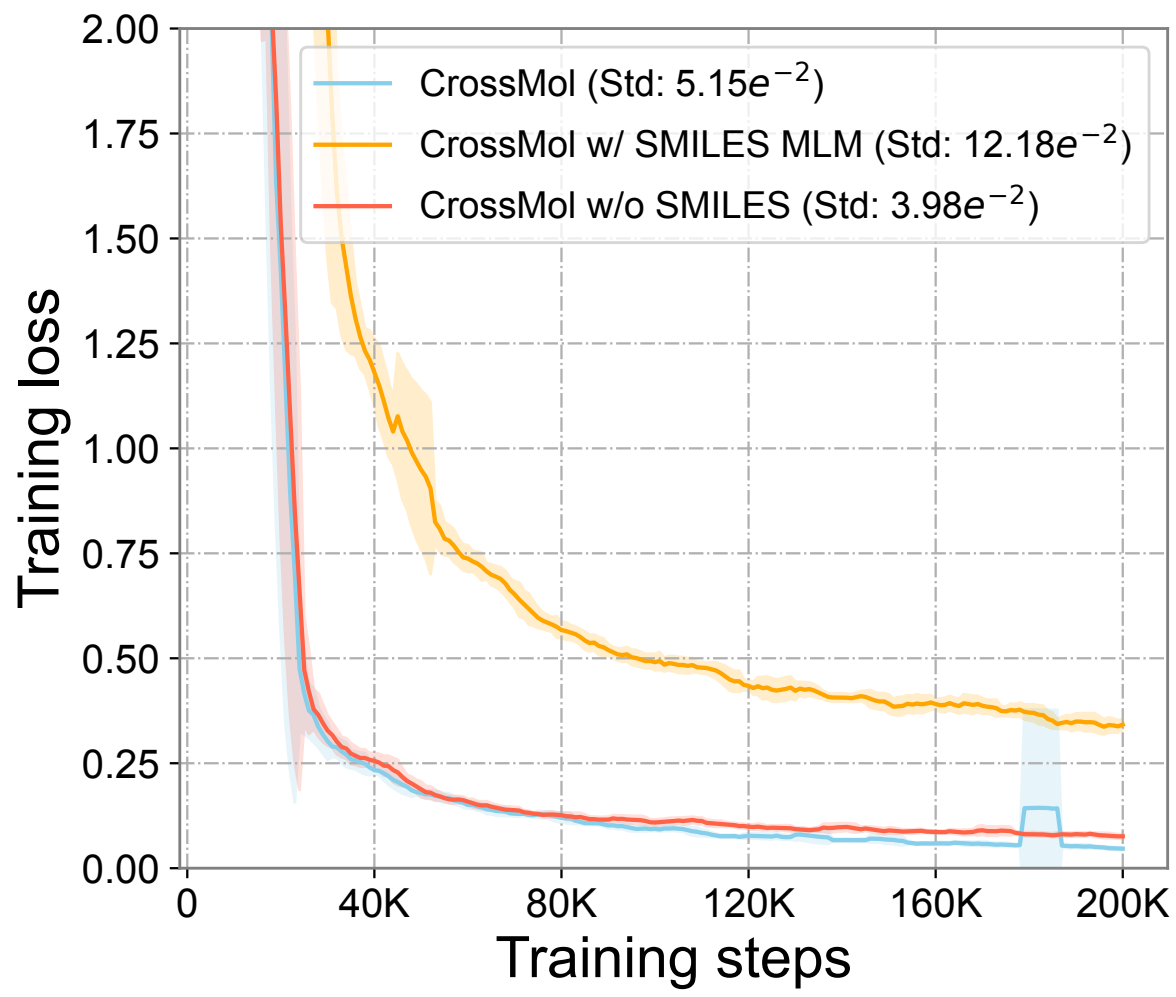

(a) Ablations on 1D Information

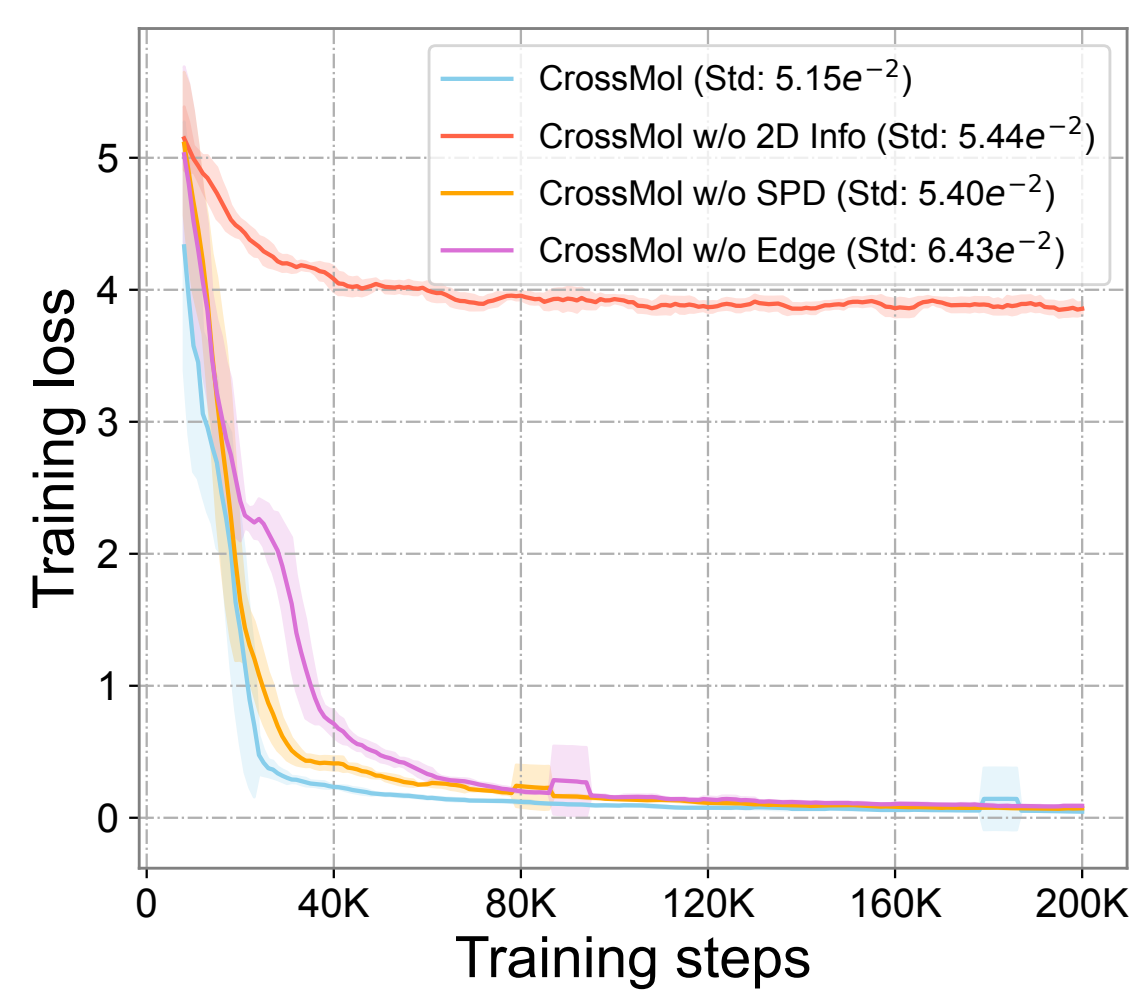

(b) Ablations on 2D Information

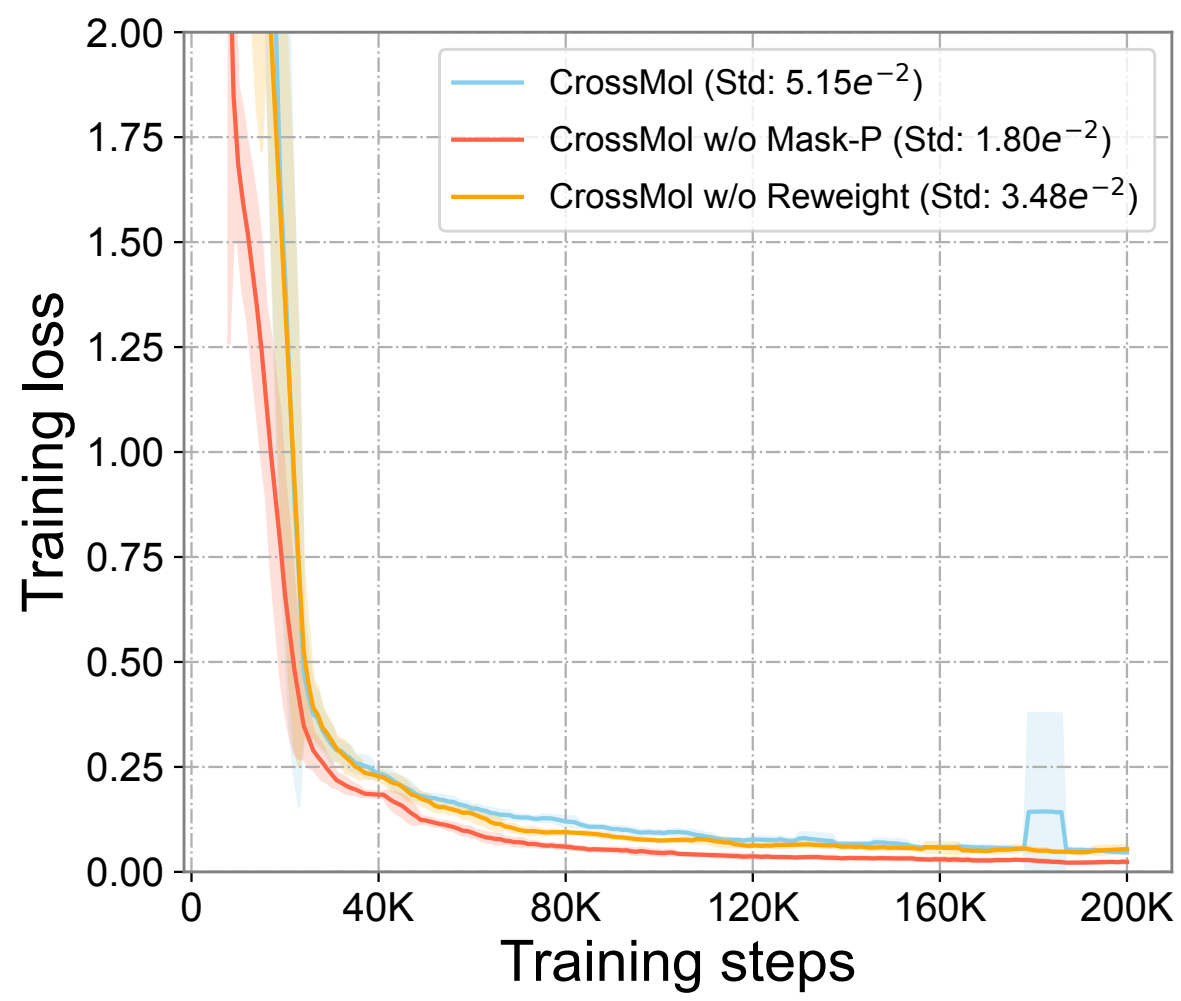

(c) Ablations on Pre-training Objectives

Figure 1: Pre-training Loss Curves of Ablation Studies on Multimodal Input and Pre-training Objectives.

Table 9: Performance Comparison under Different Dataset Sizes.

Method	Dataset Size: 10%				Dataset Size: 20%			
	BACE ↑	HIV ↑	SIDER ↑	ToxCast ↑	BACE ↑	HIV ↑	SIDER ↑	ToxCast ↑
Uni-Mol	**76.9**	73.6	55.4	59.8	80.3	78.3	58.7	62.7
CrossMol	76.6	**75.4**	**59.0**	**61.5**	**82.2**	**79.2**	**63.1**	**64.2**

References

Feng, S.; Ni, Y.; Li, M.; Huang, Y.; Ma, Z.-M.; Ma, W.-Y.; and Lan, Y. 2024. Unicorn: A unified contrastive learning approach for multi-view molecular representation learning. *arXiv preprint arXiv:2405.10343*.

Halgren, T. A. 1996. Merck molecular force field. I. Basis, form, scope, parameterization, and performance of MMFF94. *Journal of computational chemistry*, 17(5-6): 490–519.

Kingma, D. P.; and Ba, J. 2014. Adam: A method for stochastic optimization. *arXiv preprint arXiv:1412.6980*.

Riniker, S.; and Landrum, G. A. 2015. Better informed distance geometry: using what we know to improve conformation generation. *Journal of chemical information and modeling*, 55(12): 2562–2574.

Wu, Z.; Ramsundar, B.; Feinberg, E. N.; Gomes, J.; Geniesse, C.; Pappu, A. S.; Leswing, K.; and Pande, V. 2018. MoleculeNet: a benchmark for molecular machine learning. *Chemical science*, 9(2): 513–530.

Yu, Q.; Zhang, Y.; Ni, Y.; Feng, S.; Lan, Y.; Zhou, H.; and Liu, J. 2024. Multimodal Molecular Pretraining via Modality Blending. In *The Twelfth International Conference on Learning Representations*.

Zhou, G.; Gao, Z.; Ding, Q.; Zheng, H.; Xu, H.; Wei, Z.; Zhang, L.; and Ke, G. 2023. Uni-Mol: a universal 3D molecular representation learning framework.
